# Sighing Dynamics as a Candidate Digital Biomarker for Anxiety in Daily Life Using Wearable Respiratory Monitoring: Intensive Longitudinal Study

**DOI:** 10.2196/89485

**Published:** 2026-08-07

**Authors:** Xinying Zhao, Yue Li, Lizhu Zhang, Jiafeng Wang, Anfeirea Jialin, Qi An, Yunfan Fu, Cheng Yao, Wei Deng

**Affiliations:** 1 Affiliated Mental Health Center & Hangzhou Seventh People's Hospital, School of Medicine, Zhejiang University, Tianmushan Road No.305, Hangzhou, Zhejiang, 310013, China, 86 13484036877; 2 School of Computer and Computing Science, Zhejiang University, Hangzhou, Zhejiang, China; 3 School of Mental Health, Wenzhou Medical University, Wenzhou, Zhejiang, China; 4 Liangzhu Laboratory, MOE Frontier Science Center for Brain Science and Brain-Machine Integration, State Key Laboratory of Brain-Machine Intelligence, Zhejiang University, Hangzhou, Zhejiang, China

**Keywords:** anxiety disorders, sighing, respiratory physiology, digital biomarker, ecological momentary assessment, wearable sensors, digital phenotyping, intensive longitudinal data, mobile health

## Abstract

**Background:**

Sighing has been proposed as a primary respiratory reset mechanism that is often linked to emotional regulation. However, evidence linking sighing to anxiety relies heavily on laboratory studies, which lack ecological validity. It remains unclear whether ambulatory sighing dynamics in free-living settings reflect momentary (state) symptom fluctuations or enduring (trait) pathology. Evidence from daily-life monitoring is needed to evaluate sighing dynamics as a candidate digital biomarker for anxiety disorders (ADs).

**Objective:**

This study aimed to evaluate daily-life sighing dynamics as a candidate digital biomarker of anxiety by disentangling state and trait anxiety-sigh associations and comparing these dynamics between individuals with ADs and healthy controls (HCs). A secondary objective was to assess the feasibility, signal quality, and joint data coverage of a synchronized ecological momentary assessment (EMA) and respiratory inductance plethysmography (RIP) protocol.

**Methods:**

We conducted an intensive longitudinal study integrating smartphone-based EMA with continuous RIP using a Hexoskin smart shirt. Thirty-eight adults were enrolled (n=15 with ADs; n=23 HCs) and completed four 36-hour intensive monitoring blocks distributed over 1 to 2 weeks. Participants wore the Hexoskin RIP smart shirt during each 36-hour block and completed 6 randomly timed EMA prompts per day during the daytime hours (9 AM to 9 PM). Sighs were operationally defined as breaths with tidal volume ≥2 × each participant’s median tidal volume. Each EMA entry was linked to the preceding 5-minute respiratory window, and the primary outcome was sigh proportion (sigh breaths/total breaths) per window. Multilevel generalized linear mixed models were used to analyze anxiety-sigh coupling, decomposing anxiety into within-person (state) and between-person (trait) components.

**Results:**

The analysis included 1279 synchronized psychophysiological windows from 33 participants. Five participants were excluded due to insufficient valid synchronized windows. In the primary beta-binomial model of sigh proportion, higher between-person (trait) anxiety was significantly associated with a lower overall sigh proportion (odds ratio [OR] 0.80, 95% CI 0.74‐0.87; *P*<.001), while HCs showed a lower baseline sigh probability than the anxiety disorder group (OR 0.78, 95% CI 0.65‐0.93; *P*=.005). The within-person anxiety-by-group interaction was significant (OR 1.14, 95% CI 1.03‐1.26; *P*=.01), indicating that sighing tended to increase with higher momentary anxiety in HCs but was attenuated in participants with ADs. Feasibility was high (EMA completion: 1626/2046, 79.5%; high-quality respiratory samples: 10.85/12.90 million, 84.1%; EMA-RIP linkage: 1319/1626, 81.1%).

**Conclusions:**

Daily-life sighing dynamics showed a state-trait dissociation, with reduced state-dependent coupling in ADs versus HCs, supporting sighing as a candidate digital biomarker of anxiety. The synchronized EMA-RIP protocol was feasible and yielded high-integrity real-world data.

## Introduction

### Background

Sighing has long been clinically observed in anxiety and historically regarded as a symptom of pathological states such as neuroses and chronic anxiety [1,2]. Subsequent research in anxiety disorders (ADs) has reinforced this link, demonstrating that patients with ADs generally exhibit elevated sighing compared to healthy controls (HCs) across various experimental conditions, ranging from resting states and breath-holding challenges to laboratory-induced panic and recovery [3-9]. This pattern of persistent sighing, coupled with alterations in breathing rate and variability, may reflect an underlying state of “respiratory rigidity” [8,10-12], with frequent sighing representing a compensatory attempt to restore regulatory flexibility. This compensatory view is formalized in the contemporary “psychophysiological reset” model, which posits that sighing functions as a critical homeostatic safety valve to alleviate tension and restore breathing variability [12-14]. Consistent with this model, recent studies demonstrate that sighing increases specifically during relief from stress or following sustained cognitive load [14,15].

These findings raise the possibility that sighing dynamics could provide a noninvasive window into regulatory capacity. Yet, translating these laboratory-based observations into an ecologically valid digital biomarker has been difficult. Although ambulatory studies have quantified sigh frequency, findings remain mixed, with some reporting no significant group differences in aggregate measures between patients with ADs and HCs [16,17]. One explanation is that aggregating sighs across time and contexts compresses an event-like, state-dependent phenomenon into a static count, thereby attenuating the signal of interest.

To be clinically useful in real-world monitoring, sighing needs to be examined as a dynamic, state-dependent process. Its clinical value lies not in how much one sighs on average, but in when and why sighing occurs in relation to fluctuating states of anxiety [18]. This shift in focus highlights an unresolved question: whether altered sighing in anxiety reflects a stable, trait-like deficit in respiratory flexibility (“rigidity”) or a preserved, state-sensitive regulatory response to momentary distress. Validating this state-trait dissociation is therefore essential and requires evidence on whether sighing covaries with within-person fluctuations in anxiety and whether the strength of this anxiety-sigh coupling differs between diagnostic groups. Addressing this question requires high-resolution, multimodal data that synchronize subjective experience with objective physiology in daily life.

To capture this level of granularity, researchers can integrate ecological momentary assessment (EMA) with continuous respiratory inductance plethysmography (RIP) to construct “intensive longitudinal phenotypes” that capture respiratory dynamics in naturalistic settings [19,20]. While recent interventions have used cyclic sighing for stress reduction [21], observational studies that closely align EMA with respiratory monitoring remain limited. Consequently, demonstrating the technical reliability of this multimodal pipeline is a critical step in evaluating whether the descriptive phenotype of ambulatory sighing can meet the criteria for a clinically actionable digital biomarker for anxiety.

### Objectives

This intensive longitudinal study aimed to investigate whether sighing in daily life functions as a candidate digital biomarker for anxiety. The primary objective was to examine the association of sighing dynamics with within-person and between-person anxiety variation and to assess whether the observed pattern was consistent with a putative reduction in respiratory flexibility, tentatively termed “respiratory rigidity.” A secondary objective was to assess the feasibility and data quality of synchronized EMA and continuous respiratory monitoring to support future clinical translation.

## Methods

### Study Design and Participants

This intensive longitudinal study is part of an ongoing project. For the present analysis, recruitment was conducted between June 2024 and October 2025. Patients with ADs were recruited via outpatient referrals, while HCs were enrolled from the community through digital advertisements. Inclusion and exclusion criteria are summarized in Textbox 1. Following written informed consent, participants underwent a baseline assessment and the Structured Clinical Interview for *Diagnostic and Statistical Manual of Mental Disorders* (*Fifth Edition*) (*DSM-5*), Research Version, with diagnoses confirmed by clinician consensus based on *DSM-5* criteria [22]. Initially, 42 participants were recruited. Of these, 4 participants withdrew from the study, all from the AD group, mainly because of device discomfort or competing academic or work demands. Consequently, the final cohort comprised 38 participants (n=15 with ADs and n=23 HCs).

Textbox 1. **Inclusion and exclusion criteria for study participants.**

**Inclusion criteria**

- Age 18‐60 years
- Diagnosis (anxiety disorder group): diagnosis of generalized anxiety disorder, social anxiety disorder, or panic disorder according to *Diagnostic and Statistical Manual of Mental Disorders (Fifth Edition)* criteria
- Health status (healthy control group): no current or past history of psychiatric or neurological disorders
- Psychometric thresholds
  - Anxiety disorder group: Hamilton Anxiety Rating Scale (HAMA) score ≥14 and 17-item Hamilton Depression Rating Scale (HAMD-17) score <17
  - Healthy control group: HAMA score <7 and HAMD-17 score <7
- Provision of written informed consent
- Proficiency in the Chinese language sufficient to understand study procedures and prompts

**Exclusion criteria**

- Current or past diagnosis of other severe psychiatric disorders
- Presence of acute or chronic respiratory diseases or other severe somatic illnesses
- Current use of medications known to significantly affect respiration or heart rate
- History of epilepsy or a family history of epilepsy
- Current suicidal ideation or risk of self-harm
- Cognitive impairment precluding informed consent or protocol adherence
- Inability to operate wearable devices or smartphones
- Pregnancy or lactation
- Currently participating in biofeedback, mindfulness training, or other psychotherapies targeting respiratory regulation

The ambulatory monitoring protocol was designed as 4 discrete blocks over a 1‐ to 2-week period to balance high-resolution data capture with participant burden. In each block, participants combined smartphone-based EMA with continuous wearable respiratory monitoring for approximately 36 hours (9 AM on day 1 to 9 PM on day 2). A structured close-out interview was conducted upon completion to evaluate protocol implementation.

### Ethical Considerations

This study was reviewed and approved by the Ethics Committee of Hangzhou Seventh People’s Hospital (approval number 2024/037) and was conducted in accordance with the principles of the Declaration of Helsinki. All participants provided written informed consent before enrollment after being informed of the study purpose, procedures, data collection, potential risks and benefits, confidentiality protections, and their right to withdraw at any time without penalty. Questionnaire and physiological data were deidentified before analysis. Results are reported in aggregate form without disclosing identifiable information. Participants received compensation in accordance with the approved study protocol.

### Ecological Momentary Assessment

**Figure 1.**
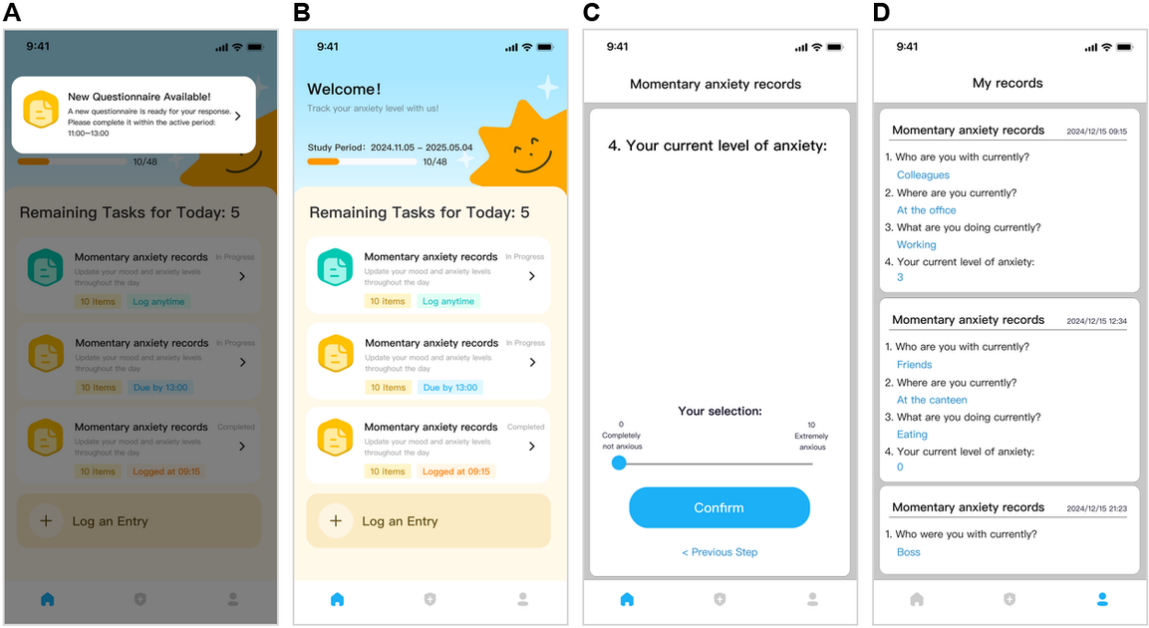
User interface of the Huixin EMAI platform. From left to right, the subfigures show (A) the push notification screen, (B) the dashboard distinguishing user-initiated (“log anytime”) and scheduled tasks, (C) the 0‐10 visual analog scale for current anxiety, and (D) the submission log. The original interface is in Chinese; English translations are provided here for illustrative purposes. Adapted with permission from ZenSeven (Hangzhou) Technology Co, Ltd.

EMA was implemented using the Huixin EMAI platform (ZenSeven Technology Co, Ltd; Figure 1) to capture real-world fluctuations in anxiety. Prior to the ambulatory phase, participants completed a supervised practice run of the app to ensure competency with the interface and protocol. We implemented a dual EMA scheme to capture high-density fluctuations in momentary states. During the active monitoring blocks, time-contingent prompts were scheduled during waking hours (9 AM to 9 PM), with the system automatically issuing a single prompt at a random time within each 2-hour interval (stratified random sampling). In parallel, participants could self-initiate entries (event-contingent sampling) at any time to capture naturally occurring anxiety episodes. At each prompt, participants reported their current situational context (location, social companions, and activity) and momentary anxiety on a visual analog scale (range: 0‐10). The present analysis focused exclusively on anxiety and situational context, although the full survey included additional exploratory items. This high-frequency, dual-sampling design maximized the capture of within-day variability in anxiety dynamics. The EMA design and reporting followed the CREMAS (Checklist for Reporting Ecological Momentary Assessment Studies) guidelines [23] (Checklist 1).

### Wearable Respiratory Monitoring

**Figure 2.**
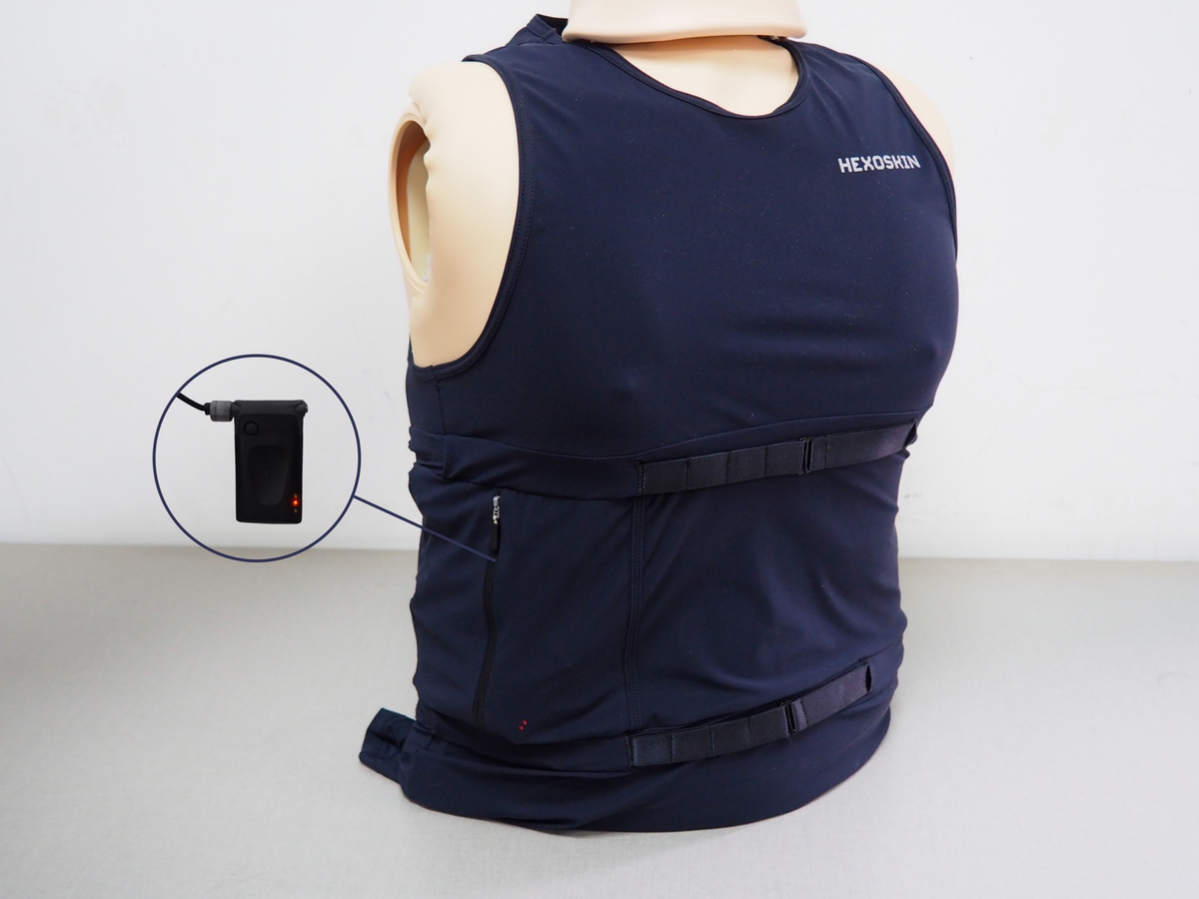
Ambulatory respiratory sensing system. The main image shows the Hexoskin ProShirt embedded with dual-band respiratory inductance plethysmography sensors. The circular inset displays the detachable Hexoskin Smart Device, which functions as the data recording module.

To acquire continuous, high-resolution respiratory waveforms in free-living settings, participants wore the Hexoskin ProShirt (Carré Technologies Inc) during the active monitoring sessions. The system comprises a machine-washable smart shirt embedded with thoracic and abdominal RIP sensors (sampled at 128 Hz) and a 3-axis accelerometer (Figure 2). Participants received standardized training on correct device fitting and were specifically instructed to moisten the textile electrodes with water or gel to optimize signal fidelity. Data collection was automated upon device connection. The dual-band RIP signals captured thoracoabdominal expansion and contraction, yielding a respiratory waveform proportional to tidal volume. This high-resolution sensing stream enabled the subsequent breath-by-breath extraction of respiratory phases and sigh events in daily life [24,25].

### Data Retention and Implementation Metrics

#### Objective Data Retention and Fidelity

To evaluate protocol feasibility, we quantified both objective data retention and user implementation. The study duration was defined as the calendar interval between the first and last day of recorded Hexoskin wear. Within this interval, valid monitoring days were categorized as EMA-active days (≥1 completed survey) or Hexoskin-active days (days with recorded respiratory data).

Regarding EMA engagement, metrics included the number of EMA-active days, the mean daily count of completed surveys, and the response latency for time-contingent prompts. The overall completion rate was calculated as the ratio of completed assessments (both scheduled and self-initiated) to the total number of scheduled prompts (expected 6 per active day). Total EMA volume was further stratified by source into scheduled versus self-initiated entries.

Respiratory data fidelity was assessed via the number of Hexoskin-active days, the mean daily duration of valid recording within the target window (9 AM to 9 PM), and wear adherence (ratio of valid recording duration to the planned 12-h window). Additionally, signal quality was indexed by the overall proportion of recorded samples that met the quality criteria [24]. Crucially for biomarker validation, joint data coverage was defined as the proportion of completed EMA events successfully linked to a valid respiratory signal in the preceding 5-minute analytic window.

#### User Experience and Acceptability

Subjective feedback was collected via a scheduled telephone debriefing interview conducted immediately upon the return of the wearable device to minimize recall bias. Research staff identified reasons for noncompliance and administered a structured 5-point Likert scale questionnaire (1=“strongly disagree,” 5=“strongly agree”). Items indexed EMA constructs (perceived burden, intrusion, adherence, usability, and privacy) and Hexoskin domains (acceptability, comfort, feasibility, privacy, social acceptance, usability, design, and perceived value).

### Data Processing

#### EMA and Respiratory Preprocessing

EMA records were restricted to the daytime monitoring window (9 AM to 9 PM). Duplicate entries submitted within 5 minutes were filtered to retain the most complete response. Device-exported respiratory data, including respiratory onset timestamps, breathing rate, tidal volume, and minute ventilation, were processed in Python (version 3.13.5) [26]. Breath cycles were defined from consecutive device-exported respiratory onset timestamps, corresponding to inspiration-to-inspiration intervals. Artifacts and extreme values were filtered using quality flags, physiological bounds, and participant-specific percentile filtering. Sighs were defined as breaths with tidal volumes exceeding twice the participant’s median tidal volume. This definition captures both regulatory sighs and high-amplitude breaths associated with daily activity [4,12].

#### Psychophysiological Synchronization

EMA and respiratory streams were synchronized on a common Unix time axis. For each EMA entry, window-level respiratory features, including breathing rate, tidal volume, minute ventilation, sigh count, and sigh proportion, were extracted from the preceding 5-minute window. This interval was selected based on sensitivity analyses (1-, 5-, 10-, and 30-min windows; see Tables S2.4 and S2.5 in Multimedia Appendix 1) as the optimal resolution for capturing sigh dynamics while minimizing noise. Only EMA entries with valid respiratory coverage in this window were linked. Participants with fewer than 15 valid linked windows were excluded to ensure stable within-person estimation [27].

### Statistical Analysis

#### Multilevel Modeling of Anxiety-Sigh Coupling

To validate sighing dynamics as a candidate state-sensitive biomarker, generalized linear mixed models were employed to quantify the psychophysiological coupling between momentary anxiety and concurrent sighing [28,29]. Because repeated EMA-linked 5-minute windows were nested within participants and the outcomes were non-Gaussian, generalized linear mixed models were used to account for within-person clustering and outcome distribution. All analyses were performed in R (version 4.3.3) using the glmmTMB package [30,31]. The primary outcome, sigh proportion, was modeled using a beta-binomial distribution (logit link) to account for overdispersion in bounded data. The secondary outcome, sigh count, was modeled using a negative binomial distribution. To disentangle state-level biomarker responses from trait-level baseline differences, anxiety scores were disaggregated into person-mean (trait) and person-mean-centered (state) components. Models specified random intercepts for participants; random slopes were excluded as they yielded negligible variance (Tables S2.1 and S2.2 in Multimedia Appendix 1). Fixed effects included within-person anxiety (state), between-person anxiety (trait), group (AD vs HC), and the critical within-person anxiety × group interaction, which tested whether the sensitivity of the biomarker differed between patients with ADs and HCs. Statistical significance was inferred from 95% CIs excluding unity.

#### Model Diagnostics and Sensitivity Analysis

Comprehensive diagnostic checks and robustness evaluations are detailed in Multimedia Appendix 1. These analyses assessed: (1) model specification and diagnostics: comparing random-intercept versus random-slope structures and evaluating residual assumptions (uniformity, dispersion, and outliers), (2) time-window robustness: re-estimating the primary models across alternative retrospective window lengths of 1, 10, and 30 minutes to verify temporal stability, (3) sensitivity to extreme windows: repeating analyses after trimming the top and bottom 0.5% of the sigh-proportion distribution to rule out potential artifacts, (4) sensitivity to baseline sociodemographic imbalance: when baseline sociodemographic characteristics differed between groups, we repeated the primary analyses with the imbalanced variable included as a participant-level covariate, and (5) mechanisms of specificity: fitting covariate-adjusted models that included contextual factors (location, activity, and social companions) and concurrent respiratory physiology (breathing rate, tidal volume, and minute ventilation) to delineate the drivers of the anxiety-sigh association.

#### Descriptive Statistics and Data Fidelity

Finally, descriptive statistics (means, SDs, and frequencies) were computed to characterize the study sample and evaluate protocol fidelity. Key metrics included adherence rates, signal quality indices, and valid joint data coverage. These steps verified the integrity of the intensive longitudinal dataset prior to biomarker modeling.

## Results

### Data Fidelity and Participant Characteristics

#### Protocol Adherence and Data Quality

High protocol adherence and signal fidelity were observed throughout the monitoring period. These metrics indicate high data fidelity for the subsequent breath-by-breath analysis. During the planned 12-hour daytime periods (9 AM to 9 PM) when EMA prompts were delivered, participants wore the smart shirt for an average of 10.97 hours, achieving 91.4% of the targeted wear time for synchronized data collection. During these periods, over 12.9 million raw respiratory samples were collected, of which 10.85 (84.1%) million met the quality criteria defined by the Hexoskin algorithm for subsequent breath-by-breath analysis. EMA compliance was high, with an overall completion rate of 79.5% (1626/2046) and a median response latency of 4.0 minutes. Of the 1626 completed EMAs, 1319 (81.1%) were successfully synchronized with valid respiratory data in the preceding 5-minute window, forming the basis for the synchronized psychophysiological analysis.

#### Analytic Sample

To ensure robust within-person estimation, the final analysis was restricted to participants contributing at least 15 valid, synchronized EMA-respiratory windows [27]. Of the 38 participants remaining after withdrawal, 5 did not meet this criterion. Missing data were handled using complete-case analysis, resulting in a final analytic sample of 33 participants (AD: n=13; HC: n=20) who contributed a total of 1279 valid psychophysiological observation windows.

#### Participant Demographics

Demographic and socioeconomic characteristics of the analytic sample are summarized in Table 1. Overall, the sample was young (mean age 28.86, SD 7.10 y) and highly educated. The participants with ADs and HCs were demographically comparable, showing no significant differences in age, sex distribution, BMI category, work status, or residence (all *P*>.05). However, a significant difference was observed in education level (*P*=.01), driven by a higher proportion of participants with postgraduate education in the HC group. Education was therefore included in sensitivity analyses as a participant-level covariate.

**Table 1.** Demographic and socioeconomic characteristics of the analytic sample (N=33).

Characteristic	AD^a^ (n=13)	HC^b^ (n=20)	Total (N=33)	*P* value^c^
Age (years), mean (SD)	33.13 (9.32)	26.08 (3.08)	28.86 (7.10)	.06
Sex, n (%)				.46
Male	5 (38.5)	5 (25)	10 (30.3)	
Female	8 (61.5)	15 (75)	23 (69.7)	
BMI category (kg/m²), n (%)				.95
Underweight (<18.5)	1 (7.7)	1 (5)	2 (6.1)	
Normal weight (18.5‐24.9)	9 (69.2)	14 (70)	23 (69.7)	
Overweight (25.0‐29.9)	3 (23.1)	5 (25)	8 (24.2)	
Education level^d^, n (%)				.01
Secondary school or below	1 (7.7)	0 (0)	1 (3)	
High school or junior college	3 (23.1)	0 (0)	3 (9.1)	
Bachelor’s degree	7 (53.8)	7 (35)	14 (42.4)	
Postgraduate degree or above	2 (15.4)	13 (65)	15 (45.5)	
Work status, n (%)				.19
Full-time employment	10 (76.9)	18 (90)	28 (84.8)	
Seeking work	1 (7.7)	2 (10)	3 (9.1)	
Not currently employed	2 (15.4)	0 (0)	2 (6.1)	
Usual residence^e^, n (%)				.99
Rural	2 (15.4)	3 (15)	5 (15.2)	
Urban	11 (84.6)	17 (85)	28 (84.8)	

a AD: anxiety disorder.

b HC: healthy control.

c Between-group differences were examined using the Mann-Whitney *U* test for continuous variables and chi-square or Fisher exact tests for categorical variables. Tests were descriptive and did not guide covariate adjustment.

d Education categories reflect the Chinese educational system but are presented here in internationally understandable terms.

e Usual residence refers to the self-reported usual place of residence.

### Descriptive Analysis of Ambulatory Data

#### Momentary Anxiety in Daily Life

Momentary anxiety ratings spanned the full range of the scale (0‐10). Although the overall intensity was low to moderate, with an overall assessment-level mean of 1.90 (SD 2.38) and a person-level mean of 2.24 (SD 2.24), the distribution indicated wide moment-to-moment variability suitable for coupling analyses. Descriptively, the participants with ADs reported higher person-level anxiety (mean 2.89, SD 2.97) compared to HCs (mean 1.81, SD 1.54; see Table S3.1 in Multimedia Appendix 2 for full details).

#### Distribution of Daily Contexts

The assessment context spanned a wide range of daily environments. Participants were most frequently sampled at their workplace (572/1279, 44.7%) or at home (485/1279, 37.9%). Regarding social context, assessments occurred most often when participants were alone (489/1279, 38.2%) or with colleagues (391/1279, 30.6%). The most reported activities were working or studying (514/1279, 40.2%), using a phone (421/1279, 32.9%), and resting (261/1279, 20.4%). This mix of active and sedentary states supports the ecological validity of the monitoring period (Table S3.2 in Multimedia Appendix 2).

#### Breathing Phenotypes in Daily Life

Regarding respiratory biomarkers, the overall mean breathing rate was 17.93 (SD 2.98) breaths per minute, and the mean tidal volume was 589.78 (SD 175.59) mL. Sighing was a frequent feature of daily breathing: on average, sighs constituted 10.88% (SD 4.41) of all breaths, with a mean count of 8.17 (SD 2.22) per 5-minute window. Descriptively, the AD group exhibited trends toward larger tidal volumes (648.79 mL vs 551.42 mL) and higher sigh proportions (11.86% vs 10.24%) compared to HCs (Table S3.3 in Multimedia Appendix 2).

### Multilevel Modeling of Anxiety-Sigh Coupling

We employed multilevel beta-binomial models to evaluate sighing dynamics as a candidate biomarker. Our analysis revealed a distinct dissociation between trait-level severity and clinical diagnosis. At the individual level, between-person anxiety was negatively associated with sigh proportion (odds ratio [OR] 0.80, 95% CI 0.74‐0.87; *P*<.001), indicating that participants with higher average anxiety scores tended to sigh less frequently in daily life. In contrast, a significant main effect was observed for clinical diagnosis: HCs exhibited a significantly lower overall sigh probability compared to participants with ADs (OR 0.78, 95% CI 0.65‐0.93; *P*=.005; Table 2). This indicates that while greater trait anxiety severity was predictive of reduced sighing at the individual level, participants with ADs exhibited a higher baseline sigh rate compared to HCs.

Regarding state-level dynamics, the analysis revealed a significant cross-level interaction between within-person anxiety and clinical diagnosis (OR 1.14, 95% CI 1.03‐1.26; *P*=.01). Decomposition of this interaction revealed a directional divergence in respiratory regulation: whereas HCs exhibited a significant positive trend in sighing probability during moments of elevated anxiety (OR 1.07, 95% CI 1.01‐1.13; *P*=.03), participants with ADs showed a marginal negative trend (OR 0.93, 95% CI 0.86‐1.01; *P*=.10). The direction of the anxiety-sigh association differed significantly between the HCs and participants with ADs.

**Figure 3.**
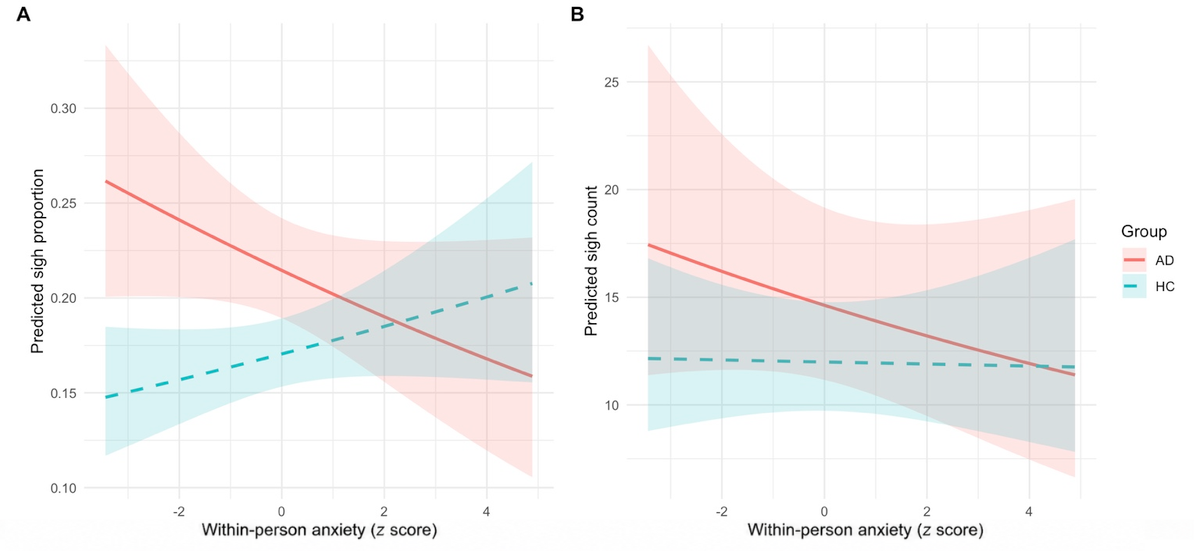
Marginal predictions illustrating the cross-level interaction between within-person anxiety (state) and diagnostic group. (A) Predicted sigh proportion from the primary beta-binomial model. (B) Predicted sigh count (per 5-min window) from the secondary negative-binomial model. The x-axis represents within-person anxiety standardized as *z* scores (0 indicates the participant’s own mean). Shaded bands represent 95% CIs. The healthy control group is depicted with dashed lines, while the anxiety disorders group is shown with solid lines. Predictions are based on random-intercept models with between-person anxiety (trait) held at the sample mean. AD: anxiety disorder; HC: healthy control.

**Figure 4.**
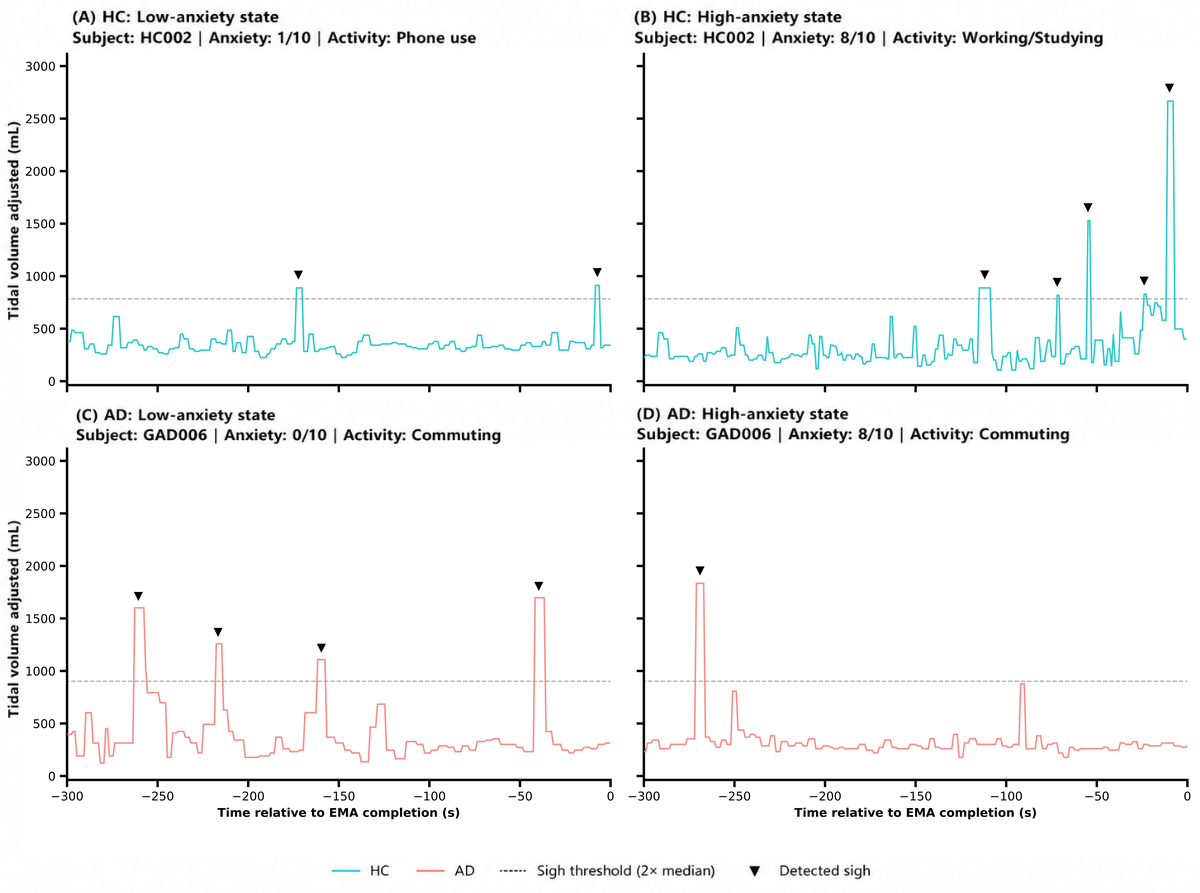
Exemplar respiratory volume traces illustrating group differences in state-dependent sigh-related respiratory patterns. (A) Healthy control (HC002) during low anxiety (1/10) while using the phone. (B) Healthy control (HC002) during high anxiety (8/10) while working or studying. (C) Participant with anxiety disorder (GAD006) during low anxiety (0/10) while commuting. (D) Participant with anxiety disorder (GAD006) during high anxiety (8/10) while commuting. All traces represent adjusted total volume time-locked to the completion of the ecological momentary assessment. Arrows indicate breaths exceeding twice the participant’s median tidal volume (horizontal dashed line), operationally defined as sighs. Panel B shows more sigh-like breaths than panel A, whereas panel D shows fewer sighs and relatively stable tidal volume during the high-anxiety state compared with panel C. AD: anxiety disorder; EMA: ecological momentary assessment; HC: healthy control.

The group-level divergence is captured in marginal predictions (Figure 3; see Table 2 for full model parameters) and is further illustrated at the individual level by exemplar respiratory traces (Figure 4). Figure 4 presents matched low- and high-anxiety states for 1 HC (HC002; panels A and B) and 1 participant with an AD (GAD006; panels C and D). In the HC, the transition to high anxiety was characterized by an increased frequency of sigh-like respiratory resets compared to the low-anxiety state (compare panels A and B). Conversely, the participant with AD exhibited less flexible respiratory adjustment: despite the transition to a high-anxiety state within the same context (commuting), sigh frequency decreased, and tidal volume remained relatively stable (compare panels C and D). This contrast descriptively illustrates a divergent, state-dependent respiratory response between the clinical and control groups.

For the secondary outcome of sigh count (panel B), the negative effect of trait anxiety persisted (incidence rate ratio 0.83, 95% CI 0.78‐0.89; *P*<.001), consistent with the primary model. However, unlike the findings for sigh proportion, the momentary cross-level interaction between within-person anxiety and group did not reach statistical significance (incidence rate ratio 1.08, 95% CI 0.98‐1.18; *P*=.11), indicating that the group-dependent divergence was specific to sigh proportion rather than absolute counts. Full model parameters for both outcomes are summarized in Table 2.

**Table 2.** Fixed effects from generalized linear mixed models testing daily-life anxiety-sigh coupling (random-intercept specification)^a^.

Term	b^b^ (SE)	OR^c^/IRR^d^ (95% CI)	*P* value
Sigh proportion (beta-binomial model)^e^			
Intercept	−1.98 (0.07)	0.14 (0.12‐0.16)	<.001
Within-person anxiety (state)	−0.07 (0.04)	0.93 (0.86‐1.01)	.10
Between-person anxiety (trait)	−0.22 (0.05)	0.80 (0.74‐0.87)	<.001
Group (healthy control)^f^	−0.25 (0.09)	0.78 (0.65‐0.93)	.005
Within-person anxiety × group^g^	0.13 (0.06)	1.14 (1.03‐1.26)	.01
Sigh count (negative-binomial model)^h^			
Intercept	2.15 (0.06)	8.61 (7.64‐9.70)	<.001
Within-person anxiety (state)	−0.05 (0.04)	0.95 (0.88‐1.02)	.15
Between-person anxiety (trait)	−0.18 (0.04)	0.83 (0.78‐0.89)	<.001
Group (healthy control)^f^	−0.09 (0.08)	0.91 (0.78‐1.06)	.22
Within-person anxiety × group	0.07 (0.05)	1.08 (0.98‐1.18)	.11

a Models included random intercepts for participants. Anxiety predictors were standardized (*z* scores). The within-person term reflects momentary deviation from a participant’s own mean anxiety level (state), and the between-person term reflects the participant’s mean anxiety across the study (trait).

b b: unstandardized regression coefficient.

c OR: odds ratio. Applicable to “Sigh proportion” rows.

d IRR: incidence rate ratio. Applicable to “Sigh count” rows.

e For sigh proportion (beta-binomial model), the outcome is the proportion of breaths classified as sighs.

f The reference group for Group is participants with anxiety disorders.

g This interaction term indicates whether the within-person anxiety-sigh association differs between healthy controls and the participants with anxiety disorders.

h For sigh count (negative-binomial model), the outcome is the sigh count per 5-minute window (without offset normalization).

### Sensitivity and Robustness Analyses

#### Temporal Stability and Resistance to Artifacts

Sensitivity analyses supported the finding that the state-trait dissociation was most pronounced at the 5-minute window. Sensitivity models showed that the effect size diminished at very short (1-min) intervals and dissipated at aggregated (30-min) intervals (Tables S2.4 and S2.5 in Multimedia Appendix 1), indicating that the anxiety-sigh coupling operates primarily as a momentary regulatory phenomenon. Furthermore, the key interaction effect persisted even after trimming extreme respiratory outliers (top and bottom 0.5% of windows), suggesting that the findings were driven by systematic physiological trends rather than signal artifacts (Table S2.6 in Multimedia Appendix 1). Education-adjusted sensitivity analyses attenuated the primary effects, although coefficient directions remained broadly consistent (Table S2.7 in Multimedia Appendix 1).

#### Exploration of Alternative Explanations

Sequential adjustment models were used to examine whether the observed association could be partly accounted for by momentary context and concurrent respiratory parameters. First, controlling for momentary context (activity, location, and social companions) substantially attenuated the anxiety-sigh association (Table S2.8 in Multimedia Appendix 1). The association between anxiety and sighing was no longer significant after controlling for momentary context. Second, further adjustment for concurrent respiratory parameters (breathing rate, tidal volume, and minute ventilation) rendered the direct effect of anxiety on sighing nonsignificant (Table S2.9 in Multimedia Appendix 1). These attenuations suggest that behavioral and contextual factors and concurrent respiratory features may partially explain the observed association.

### User Experience and Acceptability

The vast majority of enrolled participants (36/38, 94.7%) participated in the structured close-out interview to evaluate the acceptability of the protocol. Feedback indicated that the intensive monitoring protocol was generally well tolerated. Participants’ ratings for the interview items for the EMA app and the wearable device are detailed in Tables 3 and 4, respectively.

**Table 3.** Acceptability and experience of the ecological momentary assessment protocol (N=36)^a^.

Item	Score, mean (SD)
Perceived burden and intrusion	
I find the number of notifications from the Huixin EMAI platform bothersome.	2.69 (1.17)
I feel that completing the questionnaires interrupts my daily activities.	2.92 (1.05)
I feel that completing one questionnaire on the Huixin EMAI platform takes a long time.	2.08 (1.11)
I feel that the study duration was too long.	2.72 (1.16)
Self-reported adherence	
During the study, I completed the questionnaires on the Huixin EMAI platform for several consecutive days.	4.69 (0.58)
During the study, I filled out questionnaires on the Huixin EMAI platform 4 or more times a day.	4.61 (0.80)
Usability and privacy	
I can understand the questions in the questionnaire.	4.58 (0.77)
I can complete the daily questionnaires privately without worrying that others will see my responses.	4.58 (0.87)

a All items were rated on a 5-point Likert scale (1=”strongly disagree,” 5=”strongly agree”). Higher scores indicate stronger agreement with the statement as worded.

**Table 4.** Experience of wearing the Hexoskin smart shirt (N=36)^a^.

Item	Score, mean (SD)
Acceptability and comfort	
I can accept wearing the Hexoskin smart shirt in daily life.	3.39 (1.32)
I usually feel comfortable when wearing the Hexoskin smart shirt.	2.81 (1.28)
When wearing the Hexoskin smart shirt, it does not interfere with my daily activities.	3.44 (1.11)
Even outside of research, I would be willing to wear the Hexoskin smart shirt or similar devices long term in daily life.	2.42 (1.38)
Feasibility and adherence	
I can adhere to wearing the Hexoskin smart shirt every day during the study-specified time windows.	4.44 (0.69)
I can keep wearing the Hexoskin smart shirt during the specified time windows in daily life.	3.61 (1.40)
Privacy and social acceptance	
I do not worry about personal privacy leaks when wearing the Hexoskin smart shirt.	4.25 (1.08)
I trust the research team to properly safeguard data uploaded via the Hexoskin device.	4.72 (0.51)
I do not mind wearing the Hexoskin smart shirt in public.	4.03 (1.11)
Usability and design	
I find the appearance/design of the Hexoskin smart shirt acceptable.	4.00 (0.89)
The instructions and operating procedures for the Hexoskin smart shirt are clear and easy to understand.	4.31 (0.89)
I did not encounter difficulties connecting the Hexoskin terminal device with the shirt.	4.42 (0.77)
Perceived value	
Wearing the Hexoskin smart shirt has positive significance for advancing scientific research.	4.39 (0.84)
I would be willing to participate in similar wearable-device studies in the future.	4.17 (1.03)

a All items were rated on a 5-point Likert scale (1=strongly disagree, 5=strongly agree). Higher scores indicate stronger agreement with the statement as worded.

Regarding the EMA protocol, participants reported low to moderate levels of perceived burden. Ratings for notification bother (mean 2.69, SD 1.17) and interference with daily activities (mean 2.92, SD 1.05) remained around the neutral midpoint, indicating that the interruptions were generally considered manageable.

For the wearable device, results revealed a distinct trade-off between feasibility and comfort. Participants reported high confidence in their ability to adhere to the monitoring schedule (mean 4.44, SD 0.69) and acknowledged that the device did not significantly interfere with daily activities (mean 3.44, SD 1.11). However, physical comfort ratings were mixed (mean 2.81, SD 1.28), and willingness to use the device for long-term purposes outside of research was low (mean 2.42, SD 1.38). Despite these physical constraints, participants rated the scientific value of the study highly (mean 4.39, SD 0.84), suggesting that their motivation to contribute to research outweighed the physical discomfort.

Trust and privacy were rated highly across both modalities, indicating that the intensive data collection did not compromise participants’ sense of security. For the EMA component, participants reported strong perceived privacy when completing questionnaires (mean 4.58, SD 0.87). Similarly, for the wearable component, participants expressed high levels of trust in the research team’s data-safeguarding capabilities (mean 4.72, SD 0.51) and minimal concern about privacy leaks (mean 4.25, SD 1.08).

From an implementation perspective, the results supported the feasibility of the protocol. While most participants (32/36, 88.9%) reported “neutral” feelings after completing EMAs, missed prompts were primarily attributed to situational factors (eg, being busy, 25/36, 69.4%; missing notifications, 25/36, 69.4%) rather than protocol fatigue or active avoidance (eg, simply did not want to complete it, 0/36, 0%; see Table S4.1 in Multimedia Appendix 3).

## Discussion

### Principal Findings

This intensive longitudinal study provides preliminary ecological evidence supporting sighing dynamics as a candidate digital biomarker for anxiety, suggesting a potential divergence between baseline differences and state-dependent coupling. Our data show that while individuals with ADs exhibited a higher baseline sigh proportion, their capacity to mobilize adaptive respiratory resets during moments of elevated anxiety may have been attenuated. In contrast, HCs showed a lower overall sigh proportion but active mobilization in response to momentary perturbations, consistent with an adaptive function. This functional divergence underscores that the biomarker is not static sigh frequency but the dynamic coupling between anxiety and respiration. Consequently, this observed pattern may point to a hypothesized form of reduced respiratory flexibility during elevated anxiety, suggesting a potential constraint on the dynamic recruitment of homeostatic mechanisms under demand.

### Sighing Dynamics as a Candidate Digital Biomarker for Anxiety

The observed pattern of anxiety-sigh coupling supports the potential clinical validity of sighing dynamics as a candidate digital biomarker of anxiety-related respiratory regulation. Our findings point to a hypothesized pattern of reduced respiratory flexibility during elevated anxiety, which may be conceptualized as “respiratory rigidity.” Our data align with the baseline elevation observed in ADs in laboratory contexts [3,8], while offering a possible explanation for the inconsistent findings of prior ambulatory studies [3,8,16,17]. We propose that previous reliance on static aggregates may have limited sensitivity to group differences because such measures combine baseline respiratory tendencies with momentary responses to anxiety, potentially obscuring clinically relevant state-dependent regulation. This suggests that establishing clinical validity requires parsing the dynamic coupling of anxiety and respiration rather than relying on a static census of symptom frequency.

Beyond resolving these inconsistencies, the context dependence of this signal may offer a useful window into everyday regulatory processes. Unlike standard metrics such as heart rate, which can be sensitive to general arousal and context, the attenuated mobilization of sighs during high-anxiety moments is consistent with a more constrained regulatory response. In this framework, the putative “reset” role of sighing may be less dynamically recruited under distress in ADs, consistent with the psychophysiological reset model [12-14]. This pattern may also relate to broader alterations in autonomic regulation, including reduced parasympathetic flexibility [32,33]. Consequently, this pattern may help differentiate symptom severity from physiological flexibility in everyday regulation [10,12,34]. Further work is needed to determine the extent to which this pattern reflects reduced regulatory flexibility rather than differences in respiratory variability, baseline breathing patterns, or momentary behavioral and contextual factors.

From a translational perspective, this phenotype may have relevance for future clinical applications by reframing the therapeutic objective from symptom suppression toward supporting regulatory capacity. Crucially, we found that the unadjusted sighing signal may retain ecological value because it captures respiratory regulation in real-world contexts. These findings provide a preliminary rationale for exploring sighing dynamics as a potential trigger signal in future Just-in-Time Adaptive Interventions. In principle, detecting moments when sigh-related regulation appears attenuated could inform the timing of brief, low-burden practices such as cyclic sighing [21] to support parasympathetic recovery. More broadly, such systems may provide external scaffolding for self-regulation, but prospective trials are needed to establish clinical benefit, define triggering rules, and evaluate safety and acceptability in real-world deployment.

### Methodological Contribution: Validating a Multimodal Platform for Dynamic Respiratory Phenotyping

Beyond characterizing the biomarker itself, our study makes a methodological contribution by establishing and empirically evaluating a multimodal platform for capturing dynamic respiratory regulation in daily life. We show that synchronizing continuous RIP with EMA is feasible and yields breath-by-breath respiratory features with higher temporal resolution than acoustic surrogates [35] or commonly available aggregated wearable metrics [16,17]. The ecological validity of this approach is underpinned by strong protocol adherence (10.97 h/d wear time; 1626/2046, 79.5% EMA completion) and reliable signal quality (10.85/12.90 million, 84.1% usable samples), demonstrating that patterns broadly consistent with the hypothesized “respiratory rigidity” construct can be characterized in daily life, despite moderate comfort ratings. Furthermore, our work highlights practical engineering considerations for translational scaling. We synchronized server-stamped EMA entries with Hexoskin respiratory streams on a common timestamp axis established during device setup to preserve temporal fidelity in EMA-physiology linkage. Our temporal sensitivity analysis empirically establishes the 5-minute window as an effective timeframe for capturing the sigh-reset dynamic. These engineering insights support the methodological groundwork for future real-time detection and triggering pipelines, including timestamp alignment and physiologically informed window selection [20].

### Limitations

We acknowledge several methodological constraints in this study. First, the modest sample size (N=33), AD-only withdrawals, residual group imbalance, and educational differences between groups limit generalizability and group comparability. Education-adjusted sensitivity analyses attenuated the primary effects, warranting cautious interpretation and replication in larger, more demographically comparable samples across diverse anxiety subtypes [3]. In addition, ambulatory respiratory signals are inherently susceptible to daily behavioral artifacts. Although supplementary sensitivity analyses included EMA-based contextual indicators, such as self-reported recent activity, location, and social companions, objective physical activity was not quantified using accelerometer-derived measures, and our protocol lacked voice activity detection, limiting our ability to distinguish spontaneous sighs from voluntary deep breaths or speech-related respiratory changes [36]. These limitations further suggest that baseline respiratory differences and unmeasured behavioral or contextual factors may have partially contributed to the observed “respiratory rigidity” pattern. Finally, as this observational design precludes causal inference, future research using interventional designs or real-time–triggered assessments is required to clarify the temporal directionality of the association between sighing dynamics and momentary anxiety, as well as their role in anxiety regulation and recovery.

### Conclusions

This intensive longitudinal study provides preliminary ecological evidence supporting sighing dynamics as a candidate digital biomarker for daily-life anxiety. We observed a state-dependent respiratory pattern consistent with a hypothesized “respiratory rigidity” phenotype, in which individuals with ADs showed attenuated mobilization of sigh-like resets during moments of elevated anxiety. These findings may inform future research on interventions that aim not only to reduce symptoms but also to support flexible physiological regulation in daily life. Methodologically, the successful implementation of high-resolution wearable respiratory monitoring supports the feasibility of capturing granular respiratory regulation in real-world settings and provides a technical basis for future precision mobile health work. Replication studies using the same timestamp alignment strategy and 5-minute windowing scheme are needed to confirm the robustness of the observed state-trait dissociation across longer monitoring periods, broader anxiety phenotypes, and samples with better-balanced sociodemographic characteristics.

## Supplementary material

10.2196/89485 Multimedia Appendix 1 Model diagnostics, robustness, and mechanistic pathways.

10.2196/89485 Multimedia Appendix 2 Supplementary descriptive statistics.

10.2196/89485 Multimedia Appendix 3 Supplemental user experience data.

10.2196/89485 Checklist 1 CREMAS-adapted checklist for ecological momentary assessment reporting.

## Abbreviations

AD: anxiety disorder
CREMAS: Checklist for Reporting Ecological Momentary Assessment Studies
*DSM-5*: *Diagnostic and Statistical Manual of Mental Disorders, Fifth Edition*
EMA: ecological momentary assessment
HC: healthy control
OR: odds ratio
RIP: respiratory inductance plethysmography
