# Supplementary material for: Sighing Dynamics as a Candidate Digital Biomarker for Anxiety in Daily Life Using Wearable Respiratory Monitoring: Intensive Longitudinal Study
Source: JMIR Form Res. 2026 Aug 7;10:e89485. doi: 10.2196/89485 (PMC13449976; doi:10.2196/89485)
Supplement: Supplementary file 1 [file formative-v10-e89485-s001.docx]

## **Multimedia Appendix 1**

This section reports comprehensive diagnostic checks and sensitivity analyses for the primary anxiety–sigh coupling models. The analyses are organized into four sections: (1) model specification and residual diagnostics; (2) robustness to temporal window length; (3) sensitivity to extreme windows (artifacts); and (4) dissection of mechanistic pathways via covariate adjustment.

### **2.1. Model Specification and Diagnostics**

This section summarizes model specification choices and diagnostic checks for the anxiety–sigh coupling analyses.

We first compared random-intercept (RI) models with random-slope (RS) specifications that included a random slope for the within-person anxiety component. RS models converged but yielded near-zero random-slope variances and boundary correlations, suggesting minimal between-subject heterogeneity in the state-level coupling. Fixed-effect estimates were highly consistent between RI and RS models for both the primary beta–binomial outcome (sigh proportion) and the secondary negative-binomial outcome (sigh count). On this basis, RI models were retained as the primary specification. RI–RS comparisons for the primary and secondary outcomes are summarised in Table S2.1 (odds ratios) and Table S2.2 (incidence rate ratios), respectively.

**Table S2.1.** Random-intercept vs random-slope specifications for the primary coupling model (sigh proportion): fixed effects (*OR*).

| **Term** | **RI *OR*** | **95% *CI* (RI)** | ***P* (RI)** | **RS *OR*** | **95% *CI* (RS)** | ***P* (RS)** |
| --- | --- | --- | --- | --- | --- | --- |
| Within-person anxiety (State) | 0.93 | 0.86–1.01 | .10 | 0.94 | 0.86–1.02 | .15 |
| Group (Healthy Control) | 0.78 | 0.65–0.93 | .005 | 0.78 | 0.65–0.92 | .004 |
| Between-person anxiety (Trait) | 0.80 | 0.74–0.87 | <.001 | 0.80 | 0.74–0.87 | <.001 |
| Within-person anxiety × Group | 1.14 | 1.03–1.26 | .01 | 1.14 | 1.02–1.26 | .016 |

Abbreviations: RI, random-intercept model; *OR*, odds ratio; *CI*, confidence interval; RS, random-slope model.

**Table S2.2.** Random-intercept vs random-slope specifications for the secondary coupling model (sigh count): fixed effects (*IRR*).

| **Term** | **RI *IRR*** | **95% *CI* (RI)** | ***P* (RI)** | **RS *OR*** | **95% *CI* (RS)** | ***P* (RS)** |
| --- | --- | --- | --- | --- | --- | --- |
| Within-person anxiety (State) | 0.95 | 0.88–1.02 | .15 | 0.95 | 0.88–1.02 | .15 |
| Group (Healthy Control) | 0.91 | 0.78–1.06 | .22 | 0.91 | 0.78–1.06 | .22 |
| Between-person anxiety (Trait) | 0.83 | 0.78–0.89 | <.001 | 0.83 | 0.78–0.89 | <.001 |
| Within-person anxiety × Group | 1.08 | 0.98–1.18 | .11 | 1.08 | 0.98–1.18 | .11 |

Abbreviations: RI, random-intercept model; *IRR*, incidence rate ratio; *CI*, confidence interval; RS, random-slope model.

Model fit was evaluated using DHARMa simulated residual diagnostics with 500 simulations per model. For the beta–binomial models, residual uniformity tests were statistically significant and outlier tests indicated a small number of extreme windows, whereas dispersion tests were not significant. Given the discrete proportional outcome and the sample size, such Kolmogorov–Smirnov deviations are expected and were concentrated in a few high-leverage windows. For the negative binomial models, there was no evidence of over- or underdispersion or outlier inflation. DHARMa diagnostic plots and test statistics are presented in Figure S2.1 and Table S2.3.

**Figure S2.1 DHARMa residual diagnostics for the primary and secondary coupling models.** (A) Primary beta–binomial random-intercept model predicting sigh proportion. (B) Primary beta–binomial random-slope model predicting sigh proportion. (C) Secondary negative-binomial random-intercept model predicting sigh count (per 5-minute window). (D) Secondary negative-binomial random-slope model predicting sigh count (per 5-minute window).

**Table S2.3.** DHARMa residual diagnostics for primary and secondary coupling models

| **Model** | **KS uniformity *P*** | **Dispersion *P*** | **Outlier *P*** |
| --- | --- | --- | --- |
| Primary RI | 3.6e-7 | 0.184 | <0.001 |
| Primary RS | 2.5e-7 | 0.172 | <0.001 |
| Secondary RI | 0.049 | 0.880 | 0.700 |
| Secondary RS | 0.145 | 0.972 | 0.460 |

Abbreviations: KS, Kolmogorov-Smirnov test; RI, random-intercept model; RS, random-slope model.

### **2.2. Time-Window Robustness**

To assess sensitivity to the temporal aggregation of respiratory data, we re-estimated the random-intercept coupling models using alternative retrospective windows of 1, 5, 10, and 30 minutes preceding each EMA prompt.

In the beta–binomial models of sigh proportion, the between-person anxiety effect was consistently negative and statistically significant across all window lengths. The within-person anxiety × group interaction was positive across windows, reaching statistical significance at 5 minutes and approaching significance at 10 minutes, but attenuating at 1 minute and becoming negligible at 30 minutes. Full estimates are reported in Table S2.4 (sigh proportion) and Table S2.5 (sigh count).

**Table S2.4.** Primary beta–binomial model: robustness to retrospective window length (sigh proportion)^a^

|  | **1-min** |  | **5-min** |  | **10-min** |  | **30-min** |  |
| --- | --- | --- | --- | --- | --- | --- | --- | --- |
| **Term** | **OR^b^** | ***P* value** | ***OR*** | ***P* value** | ***OR*** | ***P* value** | ***OR*** | ***P* value** |
| Intercept | 0.22 | <.001 | 0.27 | <.001 | 0.25 | <.001 | 0.22 | <.001 |
| Within-person anxiety (State) | 0.99 | .84 | 0.93 | .10 | 0.96 | .36 | 1.02 | .53 |
| Group (Healthy Control)^c^ | 0.80 | .14 | 0.75 | .005 | 0.81 | .01 | 0.86 | .05 |
| Between-person anxiety (Trait) | 0.70 | <.001 | 0.81 | <.001 | 0.85 | <.001 | 0.88 | <.001 |
| Within-person anxiety × Group | 1.09 | .27 | 1.13 | .03 | 1.11 | .06 | 1.01 | .87 |

Abbreviations: *OR*, odds ratio.

^a^Within-person anxiety and between-person anxiety denote the z-scored state (deviation from the participant’s mean) and trait (participant’s mean) components of EMA anxiety, respectively.
^b^ORs are exponentiated fixed-effect estimates from beta–binomial random-intercept generalized linear mixed models predicting the proportion of sigh breaths per window.
^c^Reference group for Group is AD.

**Table S2.5.** Secondary negative-binomial model: robustness to retrospective window length (sigh count) ^a^

|  | **1-min** |  | **5-min** |  | **10-min** |  | **30-min** |  |
| --- | --- | --- | --- | --- | --- | --- | --- | --- |
| **Term** | ***IRR*** | ***P* value** | ***IRR*** | ***P* value** | ***IRR*** | ***P* value** | ***IRR*** | ***P* value** |
| Intercept^b^ | 2.49 | <.001 | 14.63 | <.001 | 29.05 | <.001 | 82.00 | <.001 |
| Within-person anxiety (State) | 1.04 | .56 | 0.95 | .30 | 0.94 | .21 | 1.01 | .78 |
| Group (Healthy Control)^c^ | 0.79 | .22 | 0.82 | .26 | 0.83 | .31 | 0.85 | .39 |
| Between-person anxiety (Trait) | 0.78 | .005 | 0.85 | .03 | 0.88 | .09 | 0.94 | .44 |
| Within-person anxiety × Group | 0.98 | .82 | 1.05 | .44 | 1.10 | .10 | 0.97 | .52 |

Abbreviations: *IRR*, incidence rate ratio.

^a^IRRs are exponentiated fixed-effect estimates from negative-binomial random-intercept generalized linear mixed models predicting sigh count.
^b^No offset was applied; therefore, intercepts reflect the baseline sigh count specific to each window duration.
^c^Reference group for Group is AD.

### **2.3. Sensitivity to Extreme Windows**

To examine the influence of extreme respiratory windows (potential artifacts), we re-estimated the random-intercept models after excluding EMA-linked windows in the top and bottom 0.5% of the sigh-proportion distribution. The main fixed-effect pattern was unchanged: the negative between-person anxiety effect was very similar to the original estimates, and the within-person anxiety × group interaction remained positive and statistically reliable. Original versus trimmed estimates are reported in Table S2.6.

**Table S2.6** Sensitivity to extreme windows (random-intercept models).^a^

|  | **Primary** |  | **Secondary** |  |
| --- | --- | --- | --- | --- |
| **Effect** | **Original (*OR*)** | **Trimmed(*OR*)** | **Original(*IRR*)** | **Trimmed (*IRR*)** |
| Intercept | 0.14 | 0.13 | 8.61 | 8.57 |
| Within-person anxiety (State) | 0.93 | 0.94 | 0.95 | 0.95 |
| Group (Healthy Control) | 0.78 | 0.77 | 0.91 | 0.91 |
| Between-person anxiety (Trait) | 0.80 | 0.80 | 0.83 | 0.83 |
| Within-person anxiety × Group | 1.14 | 1.14 | 1.08 | 1.08 |

Abbreviations: *OR*, odds ratio; *IRR*, incidence rate ratio.

^a^Extreme windows were defined as the top and bottom 0.5% of the sigh-proportion distribution across EMA-linked windows. Values are ORs for the Primary model (sigh proportion) or IRRs for the Secondary model (sigh count), obtained by exponentiating fixed-effect estimates from random-intercept models before (Original) and after (Trimmed) excluding extreme windows.

### **2.4 Sensitivity to Baseline Sociodemographic Imbalance**

After adjustment for binary education status, the principal fixed-effect pattern remained broadly similar to the original 5-minute models, but the key effects were attenuated and no longer statistically significant; full estimates are reported in Table S2.7.

**Table S2.7.** Education-adjusted random-intercept models for sighing.^a^

| Term | *OR*^b^/*IRR*^c^ | 95% *CI*^d^ | *P* value |
| --- | --- | --- | --- |
| **Panel A. Primary beta–binomial GLMM (sigh proportion)** | *OR* |  |  |
| Intercept | 0.08 | 0.06–0.10 | <.001 |
| Within-person anxiety (State) | 0.97 | 0.85–1.11 | .674 |
| Group (Healthy Control)^e^ | 0.83 | 0.63–1.10 | .194 |
| Between-person anxiety (Trait) | 0.90 | 0.80–1.01 | .084 |
| Education level^f^ | 0.92 | 0.70–1.21 | .560 |
| Within-person anxiety × Group | 1.05 | 0.88–1.24 | .608 |
| **Panel B. Secondary negative-binomial GLMM (sigh count)** | IRR |  |  |
| Intercept | 1.99 | 1.55–2.57 | <.001 |
| Within-person anxiety (State) | 1.17 | 0.90–1.53 | .243 |
| Group (Healthy Control)^e^ | 0.88 | 0.60–1.28 | .500 |
| Between-person anxiety (Trait) | 0.92 | 0.78–1.09 | .315 |
| Education level^f^ | 1.02 | 0.70–1.47 | .927 |
| Within-person anxiety × Group | 0.77 | 0.55–1.06 | .113 |

Abbreviations: *OR*, odds ratio; *IRR*, incidence rate ratio; *CI*, confidence interval.
^a^Education was included as a binary participant-level covariate derived from the baseline demographic assessment. For sensitivity analyses, education was coded as postgraduate degree or above versus lower educational attainment.Models were estimated using the same 5-minute random-intercept specifications as in the primary analysis.
^b^*OR*s are exponentiated fixed-effect estimates from beta–binomial random-intercept generalized linear mixed models predicting the proportion of sigh breaths per window.
^c^*IRR*s are exponentiated fixed-effect estimates from negative-binomial random-intercept generalized linear mixed models predicting sigh count (total number of sighs per window).
^d^95% CIs are Wald intervals calculated on the logit scale (for ORs) or log scale (for IRRs) and back-transformed.
^e^Reference group for Group is AD.
^f^Reference category for the binary education covariate is lower educational attainment.

### **2.5. Mechanisms of Specificity: Covariate-Adjusted Models**

We next examined whether the observed anxiety–sigh coupling could be accounted for by momentary context or concurrent respiratory physiology.

First, we fitted context-adjusted models (Table S2.8) that added EMA context indicators: location, recent activity, and social companions. Under this specification, the group main effect and the within-person anxiety × group interaction were not estimable due to rank deficiency (intrinsic overlap between context and anxiety states). Activity state emerged as a dominant predictor, with “Resting” significantly suppressing sighing.

Second, we fitted respiratory-feature–adjusted models (Table S2.9) that added concurrent breathing rate, tidal volume, and minute ventilation. In these models, anxiety-related fixed effects became non-significant ($P>.05$), while respiratory covariates showed strong concurrent associations with sighing.

**Table S2.8.** Context-adjusted random-intercept models for sighing.**^a^**

| **Term** | ***OR*^b/^*IRR*^c^** | **95% *CI*^d^** | ***P* value** |
| --- | --- | --- | --- |
| **Panel A. Primary beta–binomial GLMM (sigh proportion)** | ***OR*** |  |  |
| Intercept | 0.29 | 0.10–0.83 | .02 |
| Within-person anxiety (State) | 1.01 | 0.84–1.20 | .96 |
| Between-person anxiety (Trait) | 0.95 | 0.66–1.37 | .79 |
| **Location^e^** |  |  |  |
| Workplace | 1.58 | 0.72–3.48 | .25 |
| Other | 1.28 | 0.63–2.63 | .49 |
| **Recent Activity^f^** |  |  |  |
| Resting | 0.25 | 0.08–0.76 | .015 |
| Working/Studying | 0.59 | 0.30–1.18 | .13 |
| Eating | 0.63 | 0.27–1.44 | .27 |
| Phone use | 0.99 | 0.38–2.54 | .98 |
| Other | 0.91 | 0.42–1.95 | .80 |
| **Social Companions^f^** |  |  |  |
| Alone | 0.81 | 0.44–1.46 | .48 |
| Colleagues | 0.68 | 0.40–1.15 | .15 |
| **Panel B. Secondary negative-binomial GLMM (sigh count)** | ***IRR*** |  |  |
| Intercept^g^ | 17.27 | 4.43–67.39 | <.001 |
| Within-person anxiety (State) | 1.11 | 0.91–1.36 | .30 |
| Between-person anxiety (Trait) | 0.71 | 0.47–1.08 | .11 |
| **Location** |  |  |  |
| Workplace | 3.54 | 1.49–8.38 | .004 |
| Other | 2.47 | 1.12–5.47 | .03 |
| **Recent Activity** |  |  |  |
| Resting | 0.22 | 0.06–0.85 | .03 |
| Working/Studying | 0.48 | 0.16–1.39 | .17 |
| Eating | 0.39 | 0.12–1.28 | .12 |
| Phone use | 0.54 | 0.14–2.08 | .37 |
| Other | 0.64 | 0.21–1.94 | .43 |
| **Social Companions** |  |  |  |
| Alone | 0.88 | 0.42–1.83 | .73 |
| Colleagues | 0.72 | 0.41–1.28 | .27 |

Abbreviations: *OR*, odds ratio; *IRR*, incidence rate ratio; *CI*, confidence interval.

^a^Group and interaction terms were not estimable due to rank deficiency and are omitted.
^b^ORs are exponentiated fixed-effect estimates from beta–binomial random-intercept generalized linear mixed models predicting the proportion of sigh breaths per window.
^c^IRRs are exponentiated fixed-effect estimates from negative-binomial random-intercept generalized linear mixed models predicting sigh count (total number of sighs per window).
^d^95% CIs are Wald intervals calculated on the logit scale (for ORs) or log scale (for IRRs) and back-transformed.
^e^Location is coded with Home as the reference.
^f^Activity and companion indicators are coded with “not endorsing the option” as the reference.
^g^No offset was applied; therefore, intercepts reflect the baseline sigh count per 5-minute window.

**Table S2.9.** Respiratory-feature–adjusted random-intercept models for sighing**.**^a^

| **Term** | ***OR*^b^/*IRR*^c^** | **95% *CI*^d^** | ***P* value** |
| --- | --- | --- | --- |
| **Panel A. Primary beta–binomial GLMM (sigh proportion)** | ***OR*** |  |  |
| Intercept^e^ | 0.14 | 0.08–0.23 | <.001 |
| Within-person anxiety (State) | 0.99 | 0.93–1.05 | .71 |
| Group (Healthy Control)^f^ | 1.28 | 0.66–2.47 | .46 |
| Between-person anxiety (Trait) | 0.82 | 0.62–1.08 | .17 |
| Within-person anxiety × Group | 0.98 | 0.91–1.05 | .59 |
| **Respiratory Covariates** |  |  |  |
| Mean breathing rate | 0.76 | 0.70–0.82 | <.001 |
| Mean tidal volume | 2.69 | 2.44–2.97 | <.001 |
| Mean minute ventilation | 1.44 | 1.30–1.59 | <.001 |
| **Panel B. Secondary negative-binomial GLMM (sigh count)** | ***IRR*** |  |  |
| Intercept^e^ | 8.78 | 5.76–13.38 | <.001 |
| Within-person anxiety (State) | 1.01 | 0.96–1.07 | .63 |
| Group (Healthy Control)^f^ | 1.07 | 0.62–1.82 | .81 |
| Between-person anxiety (Trait) | 0.86 | 0.69–1.08 | .19 |
| Within-person anxiety × Group | 1.00 | 0.93–1.07 | .96 |
| **Respiratory Covariates** |  |  |  |
| Mean breathing rate | 0.99 | 0.91–1.08 | .79 |
| Mean tidal volume | 1.86 | 1.67–2.07 | <.001 |
| Mean minute ventilation | 1.57 | 1.40–1.76 | <.001 |

Abbreviations: *OR*, odds ratio; *IRR*, incidence rate ratio; *CI*, confidence interval.

^a^All continuous predictors (anxiety and respiratory parameters) were standardized (z-scored).
^b^ORs are exponentiated fixed-effect estimates from beta–binomial random-intercept generalized linear mixed models predicting the proportion of sigh breaths per window.
^c^IRRs are exponentiated fixed-effect estimates from negative-binomial random-intercept generalized linear mixed models predicting sigh count (total number of sighs per window).
^d^95% CIs are Wald intervals calculated on the logit scale (for ORs) or log scale (for IRRs) and back-transformed.
^e^No offset was applied; therefore, intercepts reflect the baseline sigh count per 5-minute window.
^f^Reference group for Group is AD.

These sensitivity analyses suggest that sighing is a composite digital biomarker that integrates emotional arousal, metabolic demand, and behavioral context. While statistical adjustment for these components attenuates the direct anxiety signal (reflecting their shared variance), the primary (unadjusted) model remains the most clinically relevant target for digital phenotyping, as it captures the integrated psychophysiological output useful for detection in free-living settings.
