# Supplementary material for: Sighing Dynamics as a Candidate Digital Biomarker for Anxiety in Daily Life Using Wearable Respiratory Monitoring: Intensive Longitudinal Study
Source: JMIR Form Res. 2026 Aug 7;10:e89485. doi: 10.2196/89485 (PMC13449976; doi:10.2196/89485)
Supplement: Supplementary file 2 [file formative-v10-e89485-s002.docx]

## **Multimedia Appendix 2**

This appendix reports detailed descriptive statistics for the physiological, psychological, and contextual variables characterizing the analytic sample (*N*=33 participants; 1,279 EMA windows).

**Table S3.1.** Momentary anxiety ratings in daily life.^a^

| **Level** | **Group** | ***n*** | ***Mean* (*SD*)** | ***Median* (*IQR*)^b^** |
| --- | --- | --- | --- | --- |
| **Assessment level^c^** | Overall | 1279 | 1.90 (2.38) | 1.00 (3.00) |
|  | AD | 472 | 2.29 (2.82) | 1.00 (4.00) |
|  | HC | 807 | 1.67 (2.05) | 1.00 (3.00) |
| **Person level^d^** | Overall | 33 | 2.24 (2.24) | 1.54 (2.58) |
|  | AD | 13 | 2.89 (2.97) | 1.97 (3.24) |
|  | HC | 20 | 1.81 (1.54) | 1.30 (1.95) |

^a^Anxiety was rated on a visual analog scale ranging from 0 (not at all) to 10 (extremely).

^b^IQR: Interquartile Range. ^c^Assessment-level values summarize all valid EMA-linked 5-minute windows.
^d^person-level values summarize each participant’s mean across their windows.

**Table S3.2.** EMA context distribution during daily-life prompts.

| **Context Category** | **Overall *N* (%)** | **AD *n* (%)** | **HC *n* (%)** |
| --- | --- | --- | --- |
| **Location** (Single-select) |  |  |  |
| Workplace | 572 (44.7) | 164 (34.7) | 408 (50.6) |
| Home | 485 (37.9) | 187 (39.6) | 298 (36.9) |
| Other^b^ | 222 (17.4) | 121 (25.6) | 101 (12.5) |
| **Social Companions (Multi-select)^a^** |  |  |  |
| Alone | 489 (38.2) | 156 (33.1) | 333 (41.3) |
| Colleagues | 391 (30.6) | 123 (26.1) | 268 (33.2) |
| Classmates | 172 (13.4) | 45 (9.5) | 127 (15.7) |
| Partner | 141 (11) | 110 (23.3) | 31 (3.8) |
| Patients | 87 (6.8) | 15 (3.2) | 72 (8.9) |
| Parents | 70 (5.5) | 47 (10) | 23 (2.9) |
| Customers/Clients | 46 (3.6) | 34 (7.2) | 12 (1.5) |
| Friends | 38 (3.0) | 8 (1.7) | 30 (3.7) |
| Boss | 36 (2.8) | 9 (1.9) | 27 (3.3) |
| Relatives | 29 (2.3) | 25 (5.3) | 4 (0.5) |
| Students | 18 (1.4) | 2 (0.4) | 16 (2) |
| Children | 16 (1.3) | 16 (3.4) | 0 (0) |
| Other | 61 (4.8) | 36 (7.6) | 25 (3.1) |
| **Recent Activity (Multi-select)^a^** |  |  |  |
| Working/Studying | 514 (40.2) | 121 (25.6) | 393 (48.7) |
| Phone use | 421 (32.9) | 169 (35.8) | 252 (31.2) |
| Resting | 261 (20.4) | 118 (25) | 143 (17.7) |
| Eating | 212 (16.6) | 82 (17.4) | 130 (16.1) |
| Socializing | 130 (10.2) | 59 (12.5) | 71 (8.8) |
| Commuting | 76 (5.9) | 9 (1.9) | 67 (8.3) |
| Watching TV | 60 (4.7) | 27 (5.7) | 33 (4.1) |
| Exercise | 56 (4.4) | 40 (8.5) | 16 (2) |
| Cooking | 55 (4.3) | 32 (6.8) | 23 (2.9) |
| Housework | 42 (3.3) | 32 (6.8) | 10 (1.2) |
| Personal care | 27 (2.1) | 21 (4.4) | 6 (0.7) |
| Shopping | 13 (1) | 1 (0.2) | 12 (1.5) |
| Intimacy | 1 (0.1) | 1 (0.2) | 0 (0) |
| Childcare | 0 (0) | 0 (0) | 0 (0) |
| Praying/Meditating | 0 (0) | 0 (0) | 0 (0) |
| Other | 144 (11.3) | 79 (16.7) | 65 (8.1) |

Abbreviations: AD, anxiety disorder; HC, healthy control.

^a^For multi-select items, percentages indicate the proportion of EMAs in each group endorsing the option.
^b^Detailed breakdowns of “Other” categories are aggregated for brevity.

**Table S3.3.** Breathing and sighing phenotypes in daily life.^a^

| **Phenotype** | **Overall (*N*=33)** | **AD (*n*=13)** | **HC (*n*=20)** |
| --- | --- | --- | --- |
| Breathing rate (breaths/min) | 17.93 (2.98) | 16.74 (2.49) | 18.71 (3.06) |
| Tidal volume (mL) | 589.78 (175.59) | 648.79 (151.12) | 551.42 (183.22) |
| Sigh proportion (%) | 10.88 (4.41) | 11.86 (5.43) | 10.24 (3.71) |
| Sigh count (per 5-min window) | 8.17 (2.22) | 8.38 (2.96) | 8.03 (1.65) |

Abbreviations: AD, anxiety disorder; HC, healthy control.

^a^ Values are presented as mean (*SD*). All phenotypes are person-level means calculated across valid EMA-linked 5-minute windows.
