# Supplementary material for: Sighing Dynamics as a Candidate Digital Biomarker for Anxiety in Daily Life Using Wearable Respiratory Monitoring: Intensive Longitudinal Study
Source: JMIR Form Res. 2026 Aug 7;10:e89485. doi: 10.2196/89485 (PMC13449976; doi:10.2196/89485)
Supplement: Supplementary file 3 [file formative-v10-e89485-s003.docx]

## **Multimedia Appendix 3**

**Table S4.1.** Reasons for not completing EMA prompts on time (*N*=36).^a^

| **Reason** | ***n* (%)** |
| --- | --- |
| I was too busy (work/class/other tasks). | 25 (69.4) |
| I did not see the questionnaire notification in time. | 25 (69.4) |
| I was asleep. | 12 (33.3) |
| I did not have my phone with me. | 5 (13.9) |
| I simply did not want to complete it (even without other reasons). | 0 (0) |
| I did not want others to see (privacy concern). | 1 (2.8) |
| The questionnaire took too long. | 2 (5.6) |
| My phone was unavailable, lost, or damaged. | 0 (0) |
| Others. | 3 (8.3) |

^a^Items were multiple choice; therefore, counts exceed sample size (*N*=36) due to multiple endorsements.
