# Supplementary material for: Sighing Dynamics as a Candidate Digital Biomarker for Anxiety in Daily Life Using Wearable Respiratory Monitoring: Intensive Longitudinal Study
Source: JMIR Form Res. 2026 Aug 7;10:e89485. doi: 10.2196/89485 (PMC13449976; doi:10.2196/89485)
Supplement: Supplementary file 4 [file formative-v10-e89485-s004.docx]

## **Checklist 1**

**Table S1**. CREMAS-adapted checklist for EMA reporting.

| **Section** | **Item** | | **Recommendation** | **Location in this manuscript (section / sub-section)** |
| --- | --- | --- | --- | --- |
| **Title** | | 1 | Include “ecological momentary assessment” (EMA) in the title and keywords. | Title; Keywords |
| **Introduction** | | 2 | Briefly introduce the concept of EMA and provide reasons for utilizing EMA for this study. | Introduction |
| **Methods** | | 3 | Training: Indicate whether, and by what methods, participants were trained for the EMA protocol. | Methods (Ecological Momentary Assessment; Wearable Respiratory Monitoring) |
|  | | 4 | Technology: Describe what technology was used (device, OS, app name). | Methods (Ecological Momentary Assessment; Wearable Respiratory Monitoring) |
|  | | 5 | Wave duration: State the number of EMA waves/monitoring periods. | Methods (Ecological Momentary Assessment; Wearable Respiratory Monitoring) |
|  | | 6 | Monitoring period: State the number of days each wave lasted. | Methods (Study Design and Participants) |
|  | | 7 | Prompting design: Indicate the prompting strategy (time-based, event-based). | Methods (Ecological Momentary Assessment) |
|  | | 8 | Prompt frequency: Report the intended number of prompts per day. | Methods (Ecological Momentary Assessment) |
|  | | 9 | Design features: Describe any design features intended to reduce bias or participant burden. | Methods (Study Design and Participants; Ecological Momentary Assessment) |
| **Results** | | 10 | Attrition: Indicate participant attrition throughout the study. | Results (Participant Characteristics) |
|  | | 11 | Prompt delivery: Report the number of EMA prompts planned and delivered. | Results (Data Fidelity and Participant Characteristics) |
|  | | 12 | Latency: Report the distribution of response latencies. | Results (Data Fidelity and Participant Characteristics) |
|  | | 13 | Compliance rate: Report the total number of answered EMA prompts and compliance rates. | Results (Data Fidelity and Participant Characteristics) |
|  | | 14 | Missing data: Report how missing data were handled analytically. | Results (Data Fidelity and Participant Characteristics) |
| **Discussion** | | 15 | Limitations: Discuss limitations of the study (bias, device issues). | Discussion (Limitations) |
|  | | 16 | Conclusions: Provide a general interpretation of results and benefits of using EMA. | Discussion (Conclusions) |

Table adapted from Liao Y, Skelton K, Dunton G, Bruening M. A systematic review of methods and procedures used in ecological momentary assessments of diet and physical activity research in youth: An adapted STROBE checklist for reporting EMA studies (CREMAS). J Med Internet Res. 2016;18(6): e151. <https://doi.org/10.2196/jmir.4954>.
